# Supplementary material for: Risk of colorectal cancer in patients with diabetes mellitus: A Swedish nationwide cohort study
Source: PLoS Med. 2020 Nov 13;17(11):e1003431. doi: 10.1371/journal.pmed.1003431 (PMC7665813; doi:10.1371/journal.pmed.1003431)
Supplement: S1 Table — (DOCX) [file pmed.1003431.s004.docx]

**S1 Table.** Sex and age-specific 10-year cumulative risk of colorectal cancer in population and different risk groups by personal history of diabetes (diagnosed before age 50) and family history of colorectal cancer

|  |  | **10-year cumulative risk (CR)** | | | | | | | | | | | | | | | | | | |
| --- | --- | --- | --- | --- | --- | --- | --- | --- | --- | --- | --- | --- | --- | --- | --- | --- | --- | --- | --- | --- |
|  |  | **Population** | | |  | **No DM, No FH** | | |  | | **DM Dx age <50, No FH** | | | |  | | **DM Dx age <50, FH** | | | |
| **Sex** | **Age group, y** | **N** | **%** | **95% CI** |  | **N** | **%** | **95% CI** | |  | | **N** | **%** | **95% CI** | |  | | **N** | **%** | **95% CI** |
| **Men** | **0-9** | 46 | 0.00 | 0.00–0.00 |  | 46 | 0.00 | 0.00–0.00 | |  | | 0 | 0.00 | - | |  | | 0 | 0.00 | - |
|  | **10-14** | 191 | 0.01 | 0.01–0.01 |  | 189 | 0.01 | 0.01–0.01 | |  | | 0 | 0.00 | - | |  | | 0 | 0.00 | - |
|  | **15-19** | 317 | 0.01 | 0.01–0.01 |  | 309 | 0.01 | 0.01–0.01 | |  | | 1 | 0.01 | 0.00–0.02 | |  | | 0 | 0.00 | - |
|  | **20-24** | 483 | 0.02 | 0.01–0.02 |  | 461 | 0.01 | 0.01–0.02 | |  | | 5 | 0.04 | 0.01–0.08 | |  | | 0 | 0.00 | - |
|  | **25-29** | 830 | 0.03 | 0.03–0.03 |  | 775 | 0.03 | 0.02–0.03 | |  | | 12 | 0.08 | 0.03–0.12 | |  | | 0 | 0.00 | - |
|  | **30-34** | 1,487 | 0.05 | 0.05–0.05 |  | 1,360 | 0.05 | 0.05–0.05 | |  | | 28 | 0.15 | 0.10–0.21 | |  | | 2 | 0.54 | 0.00–1.16 |
|  | **35-39** | 2,581 | 0.09 | 0.09–0.09 |  | 2,342 | 0.08 | 0.08–0.09 | |  | | 48 | 0.20 | 0.14–0.26 | |  | | 8 | 1.06 | 0.26–1.86 |
|  | **40-44** | 4,451 | 0.16 | 0.16–0.17 |  | 4,013 | 0.15 | 0.15–0.16 | |  | | 94 | 0.34 | 0.28–0.41 | |  | | 9 | 0.89 | 0.26–1.51 |
|  | **45-49** | 7,722 | 0.31 | 0.30–0.31 |  | 6,867 | 0.29 | 0.28–0.29 | |  | | 133 | 0.53 | 0.44–0.62 | |  | | 8 | 0.61 | 0.16–1.06 |
|  | **50-54** | 12,780 | 0.56 | 0.55–0.56 |  | 11,228 | 0.52 | 0.51–0.53 | |  | | 165 | 0.92 | 0.77–1.06 | |  | | 13 | 1.24 | 0.56–1.91 |
|  | **55-59** | 19,409 | 0.95 | 0.94–0.97 |  | 16,897 | 0.89 | 0.88–0.90 | |  | | 176 | 1.33 | 1.13–1.53 | |  | | 17 | 2.16 | 1.14–3.17 |
|  | **60-64** | 25,926 | 1.53 | 1.51–1.55 |  | 22,520 | 1.43 | 1.42–1.45 | |  | | 135 | 1.81 | 1.48–2.14 | |  | | 14 | 2.74 | 1.22–4.23 |
|  | **65-69** | 29,544 | 2.28 | 2.26–2.31 |  | 25,732 | 2.16 | 2.13–2.18 | |  | | 90 | 2.49 | 1.91–3.08 | |  | | 8 | 4.05 | 0.82–7.19 |
|  | **70-74** | 28,155 | 3.09 | 3.06–3.13 |  | 24,632 | 2.96 | 2.92–3.00 | |  | | 50 | 3.32 | 2.23–4.41 | |  | | 3 | 2.52 | 0.00–5.45 |
|  | **75-79** | 21,205 | 3.68 | 3.62–3.73 |  | 18,684 | 3.56 | 3.51–3.61 | |  | | 19 | 3.03 | 1.23–4.80 | |  | | 0 | 0.00 | - |
|  | **80-84** | 11,477 | 3.70 | 3.63–3.78 |  | 10,182 | 3.62 | 3.54–3.70 | |  | | 2 | 1.09 | 0.00–2.60 | |  | | 0 | 0.00 | - |
|  | **85-89** | 3,968 | 2.95 | 2.86–3.11 |  | 3,534 | 2.92 | 2.82–3.09 | |  | | 0 | 0.00 | - | |  | | 0 | 0.00 | - |
|  |  |  |  |  |  |  |  |  | |  | |  |  |  | |  | |  |  |  |
| **Women** | **0-9** | 105 | 0.00 | 0.00–0.00 |  | 104 | 0.00 | 0.00–0.00 | |  | | 0 | 0.00 | - | |  | | 0 | 0.00 | - |
|  | **10-14** | 314 | 0.01 | 0.01–0.01 |  | 310 | 0.01 | 0.01–0.01 | |  | | 1 | 0.01 | 0.00–0.03 | |  | | 0 | 0.00 | - |
|  | **15-19** | 451 | 0.02 | 0.01–0.02 |  | 439 | 0.02 | 0.01–0.02 | |  | | 4 | 0.03 | 0.00–0.07 | |  | | 1 | 1.36 | 0.00–3.97 |
|  | **20-24** | 587 | 0.02 | 0.02–0.02 |  | 561 | 0.02 | 0.02–0.02 | |  | | 5 | 0.04 | 0.01–0.08 | |  | | 1 | 1.36 | 0.00–3.97 |
|  | **25-29** | 867 | 0.03 | 0.03–0.03 |  | 818 | 0.03 | 0.03–0.03 | |  | | 4 | 0.03 | 0.00–0.06 | |  | | 0 | 0.00 | 0.00–0.00 |
|  | **30-34** | 1,549 | 0.05 | 0.05–0.06 |  | 1,444 | 0.05 | 0.05–0.05 | |  | | 8 | 0.05 | 0.02–0.09 | |  | | 1 | 0.26 | 0.00–0.77 |
|  | **35-39** | 2,755 | 0.10 | 0.09–0.10 |  | 2,548 | 0.09 | 0.09–0.10 | |  | | 17 | 0.10 | 0.05–0.14 | |  | | 2 | 0.40 | 0.00–0.99 |
|  | **40-44** | 4,646 | 0.17 | 0.17–0.18 |  | 4,253 | 0.17 | 0.16–0.17 | |  | | 42 | 0.21 | 0.15–0.28 | |  | | 5 | 0.62 | 0.07–1.16 |
|  | **45-49** | 7,564 | 0.30 | 0.29–0.31 |  | 6,880 | 0.29 | 0.28–0.30 | |  | | 78 | 0.42 | 0.33–0.52 | |  | | 8 | 0.92 | 0.28–1.55 |
|  | **50-54** | 11,613 | 0.50 | 0.49–0.50 |  | 10,545 | 0.47 | 0.47–0.48 | |  | | 92 | 0.67 | 0.54–0.81 | |  | | 7 | 0.99 | 0.30–1.67 |
|  | **55-59** | 16,219 | 0.77 | 0.76–0.78 |  | 14,665 | 0.74 | 0.72–0.75 | |  | | 83 | 0.83 | 0.65–1.01 | |  | | 10 | 1.65 | 0.61–2.67 |
|  | **60-64** | 20,686 | 1.14 | 1.13–1.16 |  | 18,530 | 1.09 | 1.07–1.11 | |  | | 76 | 1.27 | 0.97–1.58 | |  | | 11 | 2.42 | 0.85–3.96 |
|  | **65-69** | 23,805 | 1.64 | 1.62–1.66 |  | 21,068 | 1.55 | 1.53–1.57 | |  | | 57 | 1.71 | 1.22–2.19 | |  | | 5 | 1.96 | 0.15–3.73 |
|  | **70-74** | 24,060 | 2.19 | 2.16–2.21 |  | 21,182 | 2.08 | 2.05–2.11 | |  | | 29 | 2.35 | 1.34–3.35 | |  | | 1 | 0.64 | 0.00–1.89 |
|  | **75-79** | 20,525 | 2.65 | 2.62–2.69 |  | 18,131 | 2.57 | 2.53–2.60 | |  | | 14 | 2.60 | 1.03–4.15 | |  | | 0 | 0.00 | - |
|  | **80-84** | 12,889 | 2.61 | 2.56–2.65 |  | 11,422 | 2.55 | 2.50–2.60 | |  | | 4 | 7.94 | 0.00–20.0 | |  | | 0 | 0.00 | - |
|  | **85-89** | 5,037 | 1.97 | 1.91–2.04 |  | 4,462 | 1.93 | 1.87–2.01 | |  | | 1 | 6.89 | 0.00–19.1 | |  | | 0 | 0.00 | - |

Abbreviations: CR = cumulative risk; CI = confidence interval; DM = diabetes mellitus; FH = family history; Dx age = diagnosis age
