# Supplementary material for: Risk of colorectal cancer in patients with diabetes mellitus: A Swedish nationwide cohort study
Source: PLoS Med. 2020 Nov 13;17(11):e1003431. doi: 10.1371/journal.pmed.1003431 (PMC7665813; doi:10.1371/journal.pmed.1003431)
Supplement: S2 Table — (DOCX) [file pmed.1003431.s005.docx]

**S2 Table.** Comparison between recommended risk-adapted starting ages of screening in the US, Canada, and UK Guidelines and our evidence-based ones

|  |  |  | **Recommended starting age of colorectal cancer in population** | | | | | | | | | | |
| --- | --- | --- | --- | --- | --- | --- | --- | --- | --- | --- | --- | --- | --- |
|  |  |  | **45** | |  |  | **50** | |  |  | **60** | | |
| **Sex** | **FDR Dx Age,^a^ y** | **Example age^b^, y** | **United States^c^** | **Evidence^d^** | **Diff ^e^** |  | **Canada^c^** | **Evidence^d^** | **Diff ^e^** |  | **Part of United Kingdom^c^** | **Evidence^d^** | **Diff ^e^** |
| **Men** | **<45** | **43** | 33 | 31 | 2 |  | 33 | 32 | 1 |  | 55 | 39 | 16 |
|  | **45-49** | **47** | 37 | 31 | 6 |  | 37 | 32 | 5 |  | 55 | 39 | 16 |
|  | **≥50** | **60** | 40 | 31* | 9 |  | 45 | 32 | 13 |  | 60 | 39 | 21 |
| **Women** | **<45** | **43** | 33 | 31 | 2 |  | 33 | 38 | -5 |  | 55 | 45 | 10 |
|  | **45-49** | **47** | 37 | 31 | 6 |  | 37 | 38 | -1 |  | 55 | 45 | 10 |
|  | **≥50** | **60** | 40 | 31 | 9 |  | 45 | 38 | 7 |  | 60 | 45 | 15 |

FDR = first-degree relative; Dx Age = age at diagnosis in affected first-degree relative

^a^Age at diagnosis of colorectal cancer in the affected first-degree relative(s)

**^b^**Example youngest diagnosis age of each category was given to allow a head-to-head comparison between our starting ages and those in the guidelines.

**^c^**Recommended age of screening based on nation specific guidelines for individuals with one first-degree relative with colorectal cancer only (since diabetes has not been mentioned in any screening guidelines)

**^d^**The recommended evidence-based risk-adapted starting age of screening from our study in individuals with at least one affected first-degree relative and a personal history of diabetes (actually only 4 cases had >1 affected first-degree relatives + diabetes before age 50, so it can be considered as 1 affected first-degree relative + diabetes).

**^e^**Difference between the recommended starting age by guideline and our evidence-based value.

(years).

*Example: In a country with a benchmark initial screening age at 45 years, our study recommended men with one affected first-degree relative with colorectal cancer diagnosed at age 60 and a personal history of diabetes could start screening at age 31 years, whereas the US Multi-Society Task Force on Colorectal Cancer recommends that at age 40 years, nine years later.
