# Supplementary material for: Risk of colorectal cancer in patients with diabetes mellitus: A Swedish nationwide cohort study
Source: PLoS Med. 2020 Nov 13;17(11):e1003431. doi: 10.1371/journal.pmed.1003431 (PMC7665813; doi:10.1371/journal.pmed.1003431)
Supplement: S1 eDiscussion — (DOCX) [file pmed.1003431.s006.docx]

**S1 eDiscussion**

We were able to provide highly-valid evidence-based risk estimates with clinical application in the form of how many years earlier/later individuals with diabetes with/without family history of CRC attain the risk at which their counterparts in the general population are recommended to initiate CRC screening. Our findings showed that risk-adapted CRC screening by diabetes personal history with and without family history of CRC might be beneficial. Sweden is one of the few countries with high Human Development Index (HDI) without a widespread CRC screening. As a result, colorectal cancer incidence has not markedly changed in the time-frame of the study as demonstrated by NORDCAN data, suggesting effects of a potential cohort-effect are minimalized (**S3 Fig**) [1]. This makes Sweden an ideal population for such an investigation.

Our results in terms of proportion of patients with colorectal cancer who at the time of their diagnosis had a history of such a cancer in their first-degree relatives (4.3%) was in line with that in another study on risk of colorectal cancer by family history constellations (4.4%) [2]. Another Swedish study utilizing the family-cancer datasets demonstrated that risk of CRC in FDR by type of relationship (i.e. parents, siblings) did not show significant differences [3].

It is possible that age at diagnosis of the FDR with CRC in those with diabetes might affect the age of screening, however due to the limited number of young diabetic patients with a CRC family history we could not further stratify screening age by age at FDR diagnosis. Risk-adapted CRC screening ages, irrespective of diabetes status, by age of FDR diagnosis have been provided by our group in a 2020 study [4]. We also lacked data on treatment of diabetes and end-organ manifestations of diabetes. Studies have shown that insulin use, and high hemoglobin A1C (HgbA1C) levels, which represent advanced diabetes, were associated with increased CRC risk [5-7]. However, metformin is generally the first treatment option for type 2 diabetes and has a protective effect on CRC risk. A related study showed that the majority of diabetes cases with CRC before 50 in our database below the age of 50 have type 2 diabetes [8]. This suggests that any confounding affect from diabetes treatment in our findings is likely to dilute (and not over-estimate) 10-year cumulative risk values.

Awareness of an association between diabetes and CRC is fairly new making it unlikely that patients in our database would have sought colonoscopy for their increased risk. We also did not have information on families with patients with hereditary nonpolyposis colorectal cancer (HNPCC). However, we were able to identify patients who likely were based on Amsterdam II criteria which specifies an individual has to have three relatives with CRC to be tested for HNPCC. Removing these individuals did not affect CRC risk estimates in a related study using the same database [3].

Although further investigation is required into the practicality of earlier screening of diabetic patients, frequency of several shared diabetes and CRC risk factors suggest that risk estimates in our study population may in fact be conservative and risk-adapted CRC screening in diabetic patients may be even more important in other nations. The prevalence of diabetes in Sweden is approximately 4.5%, whereas in the US it is around 9% [9]. As of 2014, mean BMI in Sweden was also significantly lower (25.8) in comparison to the US (28.8) [10]. Sweden is also known to have one of the lowest age-standardized rates of smoking in Europe (18.9%) and is also considerably lower than in the US (21.9%). Furthermore, insufficient physical activity is estimated to be prevalent in roughly 23% of the Swedish population, whereas in the US the prevalence is nearly twice as high (40%) [11]. Although the aforementioned risk factors are all potential confounders in the association between diabetes and CRC, if common risk factors such as BMI, smoking, and physical inactivity are mediating the relationship between diabetes and CRC, our findings would suggest that risk of CRC in diabetic patients is likely higher in other populations. Therefore, screening diabetic patients earlier may be even more important in countries such as the US, where these risk factors are more prominent. The utility of diabetes mellitus as a risk factor would appear to depend on the availability of diabetes screening or diagnosis. Although studies have found that about 20% of patients with type 2 diabetes remain undiagnosed, due the presence of opportunistic screening for diabetes in Sweden since the 1980s, a much smaller percentage of undiagnosed cases are expected in comparison to the rest of the world [12-14].

**References in Supporting Information**

1. NORDCAN: Cancer Incidence, Mortality, Prevalence and Survival in the Nordic Countries, Version 8.2 (26.03.2019). [Internet]. Danish Cancer Society. Accessed on 23/08/2020. 2019 [cited 23/08/2020]. Available from: <http://www.ancr.nu>.

2. Tian Y, Kharazmi E, Brenner H, Xu X, Sundquist K, Sundquist J, et al. Calculating the Starting Age for Screening in Relatives of Patients With Colorectal Cancer Based on Data From Large Nationwide Data Sets. Gastroenterology. 2020;159(1):159-68 e3. Epub 2020/04/07. doi: 10.1053/j.gastro.2020.03.063. PubMed PMID: 32251666.

3. Tian Y, Kharazmi E, Sundquist K, Sundquist J, Brenner H, Fallah M. Familial colorectal cancer risk in half siblings and siblings: nationwide cohort study. BMJ (Clinical research ed). 2019;364:l803. Epub 2019/03/16. doi: 10.1136/bmj.l803. PubMed PMID: 30872356; PubMed Central PMCID: PMCPMC6417372 at [www.icmje.org/coi_disclosure.pdf](http://www.icmje.org/coi_disclosure.pdf) (available on request from the corresponding author) and declare: no support from any organisation for the submitted work (other than that described above); no financial relationships with any organisations that might have an interest in the submitted work in the previous three years; no other relationships or activities that could appear to have influenced the submitted work.

4. Tian Y, Kharazmi E, Brenner H, Xu X, Sundquist K, Sundquist J, et al. Calculating Starting Age for Screening in Relatives of Patients With Colorectal Cancer Based on Data From Large Nationwide Datasets. Gastroenterology. 2020. Epub 2020/04/07. doi: 10.1053/j.gastro.2020.03.063. PubMed PMID: 32251666.

5. Vu HT, Ufere N, Yan Y, Wang JS, Early DS, Elwing JE. Diabetes mellitus increases risk for colorectal adenomas in younger patients. World J Gastroenterol. 2014;20(22):6946-52. Epub 2014/06/20. doi: 10.3748/wjg.v20.i22.6946. PubMed PMID: 24944487; PubMed Central PMCID: PMCPMC4051936.

6. Khaw KT, Wareham N, Bingham S, Luben R, Welch A, Day N. Preliminary communication: glycated hemoglobin, diabetes, and incident colorectal cancer in men and women: a prospective analysis from the European prospective investigation into cancer-Norfolk study. Cancer Epidemiol Biomarkers Prev. 2004;13(6):915-9. Epub 2004/06/09. PubMed PMID: 15184246.

7. Siddiqui AA, Maddur H, Naik S, Cryer B. The association of elevated HbA1c on the behavior of adenomatous polyps in patients with type-II diabetes mellitus. Dig Dis Sci. 2008;53(4):1042-7. Epub 2007/10/17. doi: 10.1007/s10620-007-9970-6. PubMed PMID: 17939046.

8. Ali Khan U, Fallah M, Tian Y, Sundquist K, Sundquist J, Brenner H, et al. Personal History of Diabetes as Important as Family History of Colorectal Cancer for Risk of Colorectal Cancer: A Nationwide Cohort Study. American Journal of Gastroenterology. 2020;Publish Ahead of Print. doi: 10.14309/ajg.0000000000000669. PubMed PMID: 00000434-900000000-99302.

9. Bullard KM, Cowie CC, Lessem SE, Saydah SH, Menke A, Geiss LS, et al. Prevalence of Diagnosed Diabetes in Adults by Diabetes Type - United States, 2016. MMWR Morb Mortal Wkly Rep. 2018;67(12):359-61. Epub 2018/03/30. doi: 10.15585/mmwr.mm6712a2. PubMed PMID: 29596402; PubMed Central PMCID: PMCPMC5877361.

10. Mendis S, Davis S, Norrving B. Organizational update: the world health organization global status report on noncommunicable diseases 2014; one more landmark step in the combat against stroke and vascular disease. Stroke. 2015;46(5):e121-2. Epub 2015/04/16. doi: 10.1161/STROKEAHA.115.008097. PubMed PMID: 25873596.

11. World Health Organization. World health statistics overview 2019: monitoring health for the SDGs, sustainable development goals: World Health Organization.; 2019.

12. Andersson DK, Svardsudd K, Tibblin G. Prevalence and incidence of diabetes in a Swedish community 1972-1987. Diabet Med. 1991;8(5):428-34. Epub 1991/06/01. doi: 10.1111/j.1464-5491.1991.tb01626.x. PubMed PMID: 1830526.

13. Thunander M, Petersson C, Jonzon K, Fornander J, Ossiansson B, Torn C, et al. Incidence of type 1 and type 2 diabetes in adults and children in Kronoberg, Sweden. Diabetes Res Clin Pract. 2008;82(2):247-55. Epub 2008/09/23. doi: 10.1016/j.diabres.2008.07.022. PubMed PMID: 18804305.

14. Midthjell K, Bjorndal A, Holmen J, Kruger O, Bjartveit K. Prevalence of known and previously unknown diabetes mellitus and impaired glucose tolerance in an adult Norwegian population. Indications of an increasing diabetes prevalence. The Nord-Trondelag Diabetes Study. Scand J Prim Health Care. 1995;13(3):229-35. Epub 1995/09/01. doi: 10.3109/02813439508996766. PubMed PMID: 7481177.
