# Supplementary material for: Risk of colorectal cancer in patients with diabetes mellitus: A Swedish nationwide cohort study
Source: PLoS Med. 2020 Nov 13;17(11):e1003431. doi: 10.1371/journal.pmed.1003431 (PMC7665813; doi:10.1371/journal.pmed.1003431)
Supplement: Attachment — Submitted filename: PMEDICINE_DM_CRC screen review.docx [file pmed.1003431.s008.docx]

PMEDICINE-D-20-03051R1

Ali Khan and colleagues present their manuscript on a population level analysis of the risk of colorectal cancer in the general population and in patients with diabetes mellitus. They find that, the risk of CRC in a patient with diabetes matches the general risk when screening is normally performed about 4-5 years earlier, suggesting that patients with diabetes may benefit from earlier screening for CRC.

This is a novel analysis that provides new information on risk of CRC in the general population, and introduces the concept of risk-adapted screening for CRC. They use a large well annotated Swedish registry and also performed a sensitivity analysis excluded patients with IBD.

I found this to be overall an excellent study and novel analysis and recommend it for publication. I think the authors did a very nice job overall.

Comments:

1. It would be better to provide the actual 10-yr cumulative risk for the different categories (with confidence limits) as the first table. Table 1 could then be a separate Table 2 (with perhaps abbreviated foot notes)
2. The challenge with this type of study is to know if there are any confounders with a DM diagnosis – eg. obesity, diet, tobacco use, etc. Although the authors acknowledge this limitation in the discussion, is it possible (even if not available in the full data set) to know what the frequency of co-variates might be, and then provide a discussion around the potential impact of that?
3. The authors should be commended for doing a sensitivity analysis excluding IBD. Please provide details, including the number of patients excluded, gender, DM diagnosis.
4. Although screening begins for many at the age of 50, the reality is that the penetrance of screening is very poor. The authors suggest that they did not have access to colonoscopy data. Alternatively, could the authors provide model of 10-year risk prior to the implementation of widespread screening? The rationale for the question is that if the screening rate is high in this population, it may underestimate the impact of earlier screening for patients with DM in a population where the penetrance of screening is low.
5. The discussion should be shortened.
