# Supplementary material for: Risk of colorectal cancer in patients with diabetes mellitus: A Swedish nationwide cohort study
Source: PLoS Med. 2020 Nov 13;17(11):e1003431. doi: 10.1371/journal.pmed.1003431 (PMC7665813; doi:10.1371/journal.pmed.1003431)
Supplement: Attachment — Submitted filename: Response letter.docx [file pmed.1003431.s009.docx]

**Author Response Letter to editors and reviewers’ comments**

**Requests from the editors:**

*Please structure your abstract using the PLOS Medicine headings (Background, Methods and Findings, Conclusions - Methods and Findings should be a single subsection).

*Response: The abstract was restructured accordingly.*

*In the last sentence of the Abstract Methods and Findings section, please include a brief note about any key limitation(s) of the study's methodology.

*Response: Following sentence was added as the last sentence of the Abstract Methods and Findings section:*

“Further stratified analyses by diabetes type was not possible.”

*At this stage, we ask that you include a short, non-technical Author Summary of your research to make findings accessible to a wide audience that includes both scientists and non-scientists. The Author Summary should immediately follow the Abstract in your revised manuscript. This text is subject to editorial change and should be distinct from the scientific abstract. Please see our author guidelines for more information: <https://journals.plos.org/plosmedicine/s/revising-your-manuscript#loc-author-summary>

*Response: Following Author Summary was added immediately after the Abstract in our revised manuscript:*

“Author Summary

**Why was this study done?**

- Diabetes is associated with increased risk of colorectal cancer, especially in young adults before age 50.
- Colorectal cancer incidence is increasing among young adults who are not targeted for screening.
- Diabetes has not been considered as a risk factor in any colorectal screening guideline.

**What did the researchers do and find?**

- By analyses of information of over 12 million Swedish residents, using the world’s largest family-cancer datasets, we found how many years earlier than the general population diabetic patients with and without family history of colorectal cancer could start screening.
- Diabetic patients were observed to reach the population level of risk, at which screening starts, about 5 years earlier, and accordingly could start counseling for colorectal cancer screening earlier than the general population.
- Diabetic patients with an additional family history of colorectal cancer could start counseling for colorectal cancer screening 12-21 years earlier depending on sex and the age at which screening starts in the general population.
- When a diabetic patient younger than age 50 visits a clinic for advice on colorectal cancer screening, the clinician can easily look up the table in this study and find the suitable starting age of screening in such patients based on one simple question: Do you have a close relative (father, mother, brother, sister, son, or daughter) who has ever received a colorectal (large bowel) cancer diagnosis?

**What do these findings mean?**

- These findings for the first time provide evidence-based information about the best starting age of screening for colorectal cancer in patients with diabetes.
- A major strength of this study would relate to the extremely large and comprehensive national (Swedish) datasets available, and the duration involved (all Swedish residents born after 1931 and their parents, followed up to 2015).
- Clinicians could inform diabetic patients (with or without family history of colorectal cancer) about this possibility and encourage individualized counselling for colorectal cancer screening. ”

*Please clarify in the paper if the analytical approach reported here corresponded to one laid out in a prospective protocol or analysis plan. Please state this (either way) early in the Methods section.

a) If a prospective analysis plan (from your funding proposal, IRB or other ethics committee submission, study protocol, or other planning document written before analyzing the data) was used in designing the study, please include the relevant prospectively written document with your revised manuscript as a Supporting Information file to be published alongside your study, and cite it in the Methods section. A legend for this file should be included at the end of your manuscript.

b) If no such document exists, please make sure that the Methods section transparently describes when analyses were planned, and when/why any data-driven changes to analyses took place. 

c) In either case, changes in the analysis-- including those made in response to peer review comments-- should be identified as such in the Methods section of the paper, with rationale.

*Response: Following sentences were accordingly added to the end of Method section:*

“The main analyses were planned before starting the execution of data analyses. However, further analyses have been performed to answer reviewers’ comments, such as adding supplementary tables of basic characteristics and a table for 10-year cumulative risk by age group, with no influence on our main findings. No data-driven changes to analyses took place.”

*We'd suggest using an appropriate guideline, such as the TRIPOD guideline (for reporting of prediction models for prognosis or diagnosis) to help ensure full reporting of the methods and findings of this study - <https://www.equator-network.org/reporting-guidelines/tripod-statement/>. If using the guideline please note this and cite the guideline in your Methods section, and include a completed TRIPOD checklist as supporting information with the revised paper.

*Response: As this is not a typical study on a prediction model for prognosis or diagnosis, the use of TRIPOD guideline was not mentioned in the text. However, we reported the RECORD checklist and uploaded it.*

-----------------------------------------------------------

**Comments from the reviewers:**

Reviewer #1: "Risk-adapted colorectal cancer screening in patients with diabetes mellitus: A nationwide cohort study" employed lifetime data from over 12 million patients linked from various Swedish registers and datasets, to examine the effect of diabetes mellitus and/or family history of colorectal cancer (CRC) on an individual's ten-year cumulative risk of CRC. It was found that diabetes mellitus caused a risk advancement of about five years from the recommended screening age of fifty, compared to reference individuals without diabetes mellitus. A family history of CRC resulted in even more pronouned CRC risk advancements, of 12-21 years. This suggests that risk-adapted starting age of CRC screening may be appropriate, as summarized in Table 1.

A major strength of this study would relate to the extremely large and comprehensive national (Swedish) datasets available, and the duration involved (all Swedish residents born after 1931, followed up to 2015). Some queries however remain, mainly relating to the details of cumulative risk computation, and treatment of confounders:

1. Details on the computation of the main outcome of 10-year cumulative risk, were not found in the Methods section. To the best of our knowledge, there are various plausible ways by which cumulative risk can be estimated, together with accompanying parameters (e.g. smoothing of risk between years/within age groups). These details might be provided (possibly in supplementary data), since it appears central to the findings. If the technique used was also basically the same as relevant prior work, it might also be stated.

*Response: A description on how 10-year cumulative risks were calculated has been added to Methods section, paragraph 4, line 2:*

*“*Risk-adapted screening ages in diabetic patients were calculated using 10-year cumulative risk of CRC. The 10-year cumulative risks were calculated using the following formulas:

*•	Age-specific incidence rate = Total cases at each age (every one year) divided by the total person-years at that age*

*•	10-year cumulative rate for age X = Sum of 10 consecutive yearly age-specific incidence rates from age X to age X+9*

*•	10-year cumulative risk = 1 – exp^(−10-year cumulative rate)^*

Rather than aggregating cumulative incidence by age groups (the traditional method of calculating cumulative risk), age-specific precise values from individual participant’s yearly data were used in the calculation (1). Comparing 10-year cumulative risk in each risk groups to the population cumulative risk allowed the inference of risk-adapted screening ages. A smoothing effect to reduce random variation in incidence rates was employed using a moving average. For instance, for the 10-year cumulative risk at age 30, the 10-year cumulative risk at age 29, 30, and 31 were averaged together, while for age 31, the 10-year cumulative risk at age 30, 31 and 32 were averaged together and so on. This methods of calculating risk-adapted starting age of cancers has already been used for some other conditions (2-4).”

A related article [11] by some of the authors was briefly referenced in the discussion as the "time-dependent method in this study", but that appears to have various configurations (i.e. accumulative, static, dynamic). The authors might consider going into similar depth as [11], in describing the actual method applied, without which statistical validity is difficult to further assess.

*Response: Reference 11 (cited here as reference 5), which is a recent published work by our team, compared three different methods of ascertaining family and disease history: accumulative, static, and dynamic. The dynamic method is synonymous with ‘time-dependent’ and ‘time-varying’. We referenced this study because it shows that for the purpose of counselling and real-time risk estimation, the dynamic (time-dependent) method is most appropriate, which is the case in our current manuscript. A discussion around the validity of the dynamic method has been included in Methods, paragraph 3, line 4:*

“The rationale behind utilizing the dynamic (time-dependent) method is that it is understood to be the most appropriate for studies involving risk stratification since it provides real-time risk estimates that can be applied in clinical settings (5). For instance, if a non-diabetic individual wants to know their risk of developing CRC at the present time, only their known histories can be taken account even if they were to become diabetic later in life. Alternatively, the static (traditional method of ascertaining family and personal disease history in studies) method is possible in register-based studies where an individual’s entire prior personal or family histories are known at the conclusion of study follow-up. Resultantly, the static method would be most appropriate for estimating the effect size that a certain risk factor has on an outcome. We chose to employ the dynamic method since our primary aim was risk-adapted screening of CRC in diabetic patients that could be used for real-time counselling. Furthermore, the dynamic method reflects the time-varying nature of disease histories, making it ideal for the purposes of this study.”

2. It might be considered to also directly include relevant data (e.g. risk-adapted CRC screening ages by age of FDR diagnosis) instead of citing prior work ([26] in this case), if possible.

*Response: Thank you for this comment. Risk-adapted CRC screening ages by age of FDR diagnosis has been already published by our team as an open access article in Gastroenterology journal and is freely available (4)*. *The space limit does not allow inclusion of a very large published table that is not the main focus of the current paper. In the current work we presented the best fit age for colorectal cancer screening in diabetic patients only.*

3. The utility of diabetes mellitus as a risk factor would appear to depend on the availability of diabetes screening/diagnosis. As such, the authors might briefly discuss the prevalence/frequency/method/other characteristics of diabetes screening/diagnosis for this population, and if possible provide relevant statistics such as the observed prevalence of diabetes by age, and average onset of CRC in years after diabetes was diagnosed.

*Response: Relevant statistics of diabetic patients were added as S1 Table. As the data is nationwide, the prevalence reported in the table represent the prevalence in Sweden. The following sentences were added to Results and Discussion sections:*

*Results section, paragraph 1, line 4:*

“Among diabetic patients, the mean time to CRC diagnosis was 5.8 years.”

*eDiscussion section, last paragraph, line 15:*

“The utility of diabetes mellitus as a risk factor would appear to depend on the availability of diabetes screening or diagnosis. Although studies have found that about 20% of patients with type 2 diabetes remain undiagnosed, due the presence of opportunistic screening for diabetes in Sweden since the 1980s, a much smaller percentage of undiagnosed cases are expected in comparison to the rest of the world (6-8).”

4. All available plausible confounders (e.g. IBD, COPD?) from the registers might be systematically listed, although it is mentioned as a limitation that there was minimal access to data on lifestyle factors (e.g. obesity, recognized as a major risk factor in [9])

*Response: New supplementary tables of characteristics (S1 and S2 Tables) including the available plausible confounders (IBD, HNPCC, hospitalization for COPD, hospitalization for obesity, and hospitalization for alcoholism) and their frequencies in patients with diabetes or CRC were included and the following sentence was added to the Results section, paragraph 1, second last sentence:*

“Further characteristics of patients with diabetes and patients with CRC are presented in the Supporting Information (S1 and S2 Tables).”

5. A demographics summary table and flowchart of the subject selection procedure might be considered.

*Response: A demographic summary table of diabetic patients and CRC patients, as well as a flowchart of the final study population have been added to the supplementary data.*

*Methods, paragraph 2, second last sentence:*

“The final dataset contained maximum 51 years of follow-up from beginning of 1964 to end of 2015. A flowchart of the final study population is presented in the Supporting Information (S2 Fig).”

*Results, paragraph 1, second last sentence:*

“Further characteristics of patients with diabetes and patients with CRC are presented in the Supporting Information (S1 and S2 Tables).”

6. Minor issue: on Page 10, "did no alter those results" might be "did not".

*Response: This typo was corrected.*

-----------------------------------------------------------

Reviewer #2: This is a well performed study and the authors should be commended.

Ali Khan and colleagues present their manuscript on a population level analysis of the risk of colorectal cancer in the general population and in patients with diabetes mellitus. They find that, the risk of CRC in a patient with diabetes matches the general risk when screening is normally performed about 4-5 years earlier, suggesting that patients with diabetes may benefit from earlier screening for CRC.

This is a novel analysis that provides new information on risk of CRC in the general population, and introduces the concept of risk-adapted screening for CRC. They use a large well annotated Swedish registry and also performed a sensitivity analysis excluded patients with IBD.

I found this to be overall an excellent study and novel analysis and recommend it for publication. I think the authors did a very nice job overall.

*Response: Thank you!*

Comments:

1. It would be better to provide the actual 10-yr cumulative risk for the different categories (with confidence limits) as the first table. Table 1 could then be a separate Table 2 (with perhaps abbreviated foot notes)

*Response: Thank you for this comment. We included a table with 10-year cumulative risk of CRC for each of the risk groups (with 95% CIs) by sex and age group as S3 Table (not in the main text to avoid overlaps with content of essential Fig 1 and Fig 2). The following sentence was added to the Results section, paragraph 1, last sentence:*

“10-year cumulative risk estimates of developing CRC in diabetic patients with and without family history of colorectal cancer by sex and age group are presented in S3Table.”

2. The challenge with this type of study is to know if there are any confounders with a DM diagnosis – eg. obesity, diet, tobacco use, etc. Although the authors acknowledge this limitation in the discussion, is it possible (even if not available in the full data set) to know what the frequency of co-variates might be, and then provide a discussion around the potential impact of that?

*Response: New supplementary tables of characteristics (S1 and S2 Tables) including the available plausible confounders, such as IBD, HNPCC, hospitalization for COPD (as surrogate for heavy smoking), hospitalization for obesity (as surrogate for morbid obesity), and hospitalization for alcoholism (as surrogate for heavy alcohol consumption), and their frequencies in patients with diabetes or CRC were included and following sentences were added to the Results, Discussion and supplementary eDiscussion section.*

*Results section, paragraph 1, second last sentence:*

“Further characteristics of patients with diabetes and patients with CRC are presented in the Supporting Information (S1 and S2 Tables)

*Discussion, last paragraph, last line:*

“Further discussions around the importance of earlier screening in diabetic patients and the generalizability of our findings have been included in the supplementary eDiscussion.”

*eDiscussion, last paragraph, first line:*

“Although further investigation is required into the practicality of earlier screening of diabetic patients, frequency of several shared diabetes and CRC risk factors suggest that risk estimates in our study population may in fact be conservative and risk-adapted CRC screening in diabetic patients may be even more important in other nations. The prevalence of diabetes in Sweden is approximately 4.5%, whereas in the US it is around 9% (9). As of 2014, mean BMI in Sweden was also significantly lower (25.8) in comparison to the US (28.8) (10). Sweden is also known to have one of the lowest age-standardized rates of smoking in Europe (18.9%) and is also considerably lower than in the US (21.9%). Furthermore, insufficient physical activity is estimated to be prevalent in roughly 23% of the Swedish population, whereas in the US the prevalence is nearly twice as high (40%) (11). Although the aforementioned risk factors are all potential confounders in the association between diabetes and CRC, if common risk factors such as BMI, smoking, and physical inactivity are mediating the relationship between diabetes and CRC, our findings would suggest that risk of CRC in diabetic patients is likely higher in other populations. Therefore, screening diabetic patients earlier may be even more important in countries such as the US, where these risk factors are more prominent.”

3. The authors should be commended for doing a sensitivity analysis excluding IBD. Please provide details, including the number of patients excluded, gender, DM diagnosis.

*Response: A demographic summary table of diabetic patients and CRC patients including IBD frequency in both groups was added as S1 and S2 Tables.*

*Results, last paragraph, line 2:*

“A total of 445,444 cases of IBD (185,869 men; 44%) were excluded from the analysis. Of all IBD cases, 5,957 (1,613 men; 27%) preceded a CRC diagnosis, and 19,232 IBD cases (6751 men; 35%) were comorbid with diabetes. No substantial changes in our main estimates were detected after exclusion of IBD cases.”

4. Although screening begins for many at the age of 50, the reality is that the penetrance of screening is very poor. The authors suggest that they did not have access to colonoscopy data. Alternatively, could the authors provide model of 10-year risk prior to the implementation of widespread screening? The rationale for the question is that if the screening rate is high in this population, it may underestimate the impact of earlier screening for patients with DM in a population where the penetrance of screening is low.

*Response: S3 Fig was added providing colorectal cancer incidence trends in Sweden by age to demonstrate the lack of a cohort effect in the study population due to screening. Sweden is one of the few countries with high Human Development Index (HDI) without a widespread CRC screening. S3 Fig was added providing colorectal cancer incidence trends in Sweden by age to demonstrate the lack of a cohort effect in the study population due to availability of screening. The following sentences were added to the eDiscussion, first paragraph, line 5:*

“Sweden is one of the few countries with high Human Development Index (HDI) without a widespread CRC screening. As a result, colorectal cancer incidence has not markedly changed in the time-frame of the study as demonstrated by NORDCAN data, suggesting effects of a potential cohort-effect are minimalized (S3 Fig) (12). This makes Sweden an ideal population for such an investigation.”

5. The discussion should be shortened.

*Response: Some non-essential parts of Discussion were moved to supplementary eDiscussion.*

-----------------------------------------------------------

Reviewer #3: The authors utilized a national-wide database to provide evidence on the starting age of colorectal cancer screening considering the familial history of colorectal cancer as well as the history of diabetes. The topic is interesting and provides an important implication, however, more details for the methods and results should be provided.

Major comments

I can not find ref 26, which used the same database and addressed FDR as a determinant of starting age of colorectal cancer screening. It seems that the results of two articles overlap substantially.

*Response: The reference 26 (cited here as reference 4) was in press at the time of submission of this manuscript. We added the doi (*[*https://doi.org/10.1053/j.gastro.2020.03.063*](https://doi.org/10.1053/j.gastro.2020.03.063)*) to the reference, which is also linked to its open access full-text. There is no overlap between these two papers as that paper has nothing to do with diabetic patients.*

 
Table 1: The results are too simple. Please provide more details such as person-year, cumulative incidence at each age, etc. Why was not the concurrent DM history and family history considered? Did the "patients (Obs)" refer the cumulative numbers of colorectal patients within the groups? Please provide the results for the total population for men and women, respectively.

*Response: We have included a table of cases and 10-year cumulative risks of CRC for each of the risk groups as S3 Table.*

*The rows for ≥1 FDR in Table 1 are actually the concurrent DM history and family history. We added “Yes” to the abovementioned rows for family history in Table 1 under heading ‘Diabetes personal history’ to be clearer and avoid misunderstanding by readers.*

*We changed the explanation about Patients (Obs) in the footnote of Table 1 to be clear that these are cumulative numbers of colorectal patients within the groups:*

“CRC patients (Obs): Cumulative number of observations with colorectal cancer within the groups”

*Results for the total population for men and women are also presented in S3 Table.*

Considering the long study period, the authors may consider the period-cohort effects of colorectal cancer incidence.

*Response: S3 Fig was added providing colorectal cancer incidence trends in Sweden by age to demonstrate the lack of a cohort effect in the study population due to screening. The following sentences were added to the eDiscussion, first paragraph, line 5:*

“Sweden is one of the few countries with high Human Development Index (HDI) without a widespread CRC screening. As a result, colorectal cancer incidence has not markedly changed in the time-frame of the study as demonstrated by NORDCAN data, suggesting effects of a potential cohort-effect are minimalized (12). This makes Sweden an ideal population for such an investigation.”

Was the proportion of patients with a family history comparable with other studies conducted in Sweden? Please provide more details on the distribution of FDR, e.i. mother/father/siblings.

*Response: Although there is no previous Swedish study on personal history of diabetes and family history of CRC, our study is a nationwide cohort study and as a result all colorectal cancer studies conducted using the Swedish-family cancer datasets would have a comparable proportion of family history. Another Swedish study utilizing colorectal cancer family history data demonstrated a similar proportion of first-degree relatives with colorectal cancer (4.4%;* [*https://doi.org/10.1053/j.gastro.2020.03.063*](https://doi.org/10.1053/j.gastro.2020.03.063)*) as our study population (4.3%). Additionally, the sample size for familial cases did not allow us to provide our analyses in further strata by type of FDR, nonetheless, another study evaluating risk of CRC by FDR type showed minimal differences in risk between parents and siblings (*[*https://doi.org/10.1136/bmj.l803*](https://doi.org/10.1136/bmj.l803)*). The following sentences were added to the supplementary eDiscussion section, second paragraph:*

*“*Our results in terms of proportion of patients with colorectal cancer who at the time of their diagnosis had a history of such a cancer in their first-degree relatives (4.3%) was in line with that in another study on risk of colorectal cancer by family history constellations (4.4%) (4). Another Swedish study utilizing the family-cancer datasets demonstrated that risk of CRC in FDR by type of relationship (i.e. parents, siblings) did not show significant differences (13).”

Please provide the comparisons between the results of the current study and existing screening recommendation for the person with a family history of colorectal cancer.

*Response: We have included a comparison between our findings for diabetic patients with a CRC family history with established screening guidelines for individuals with a first-degree relative with CRC for different benchmark ages of screening. The data has been included in the supplement as S4 Table. Following sentence was added to Results section, paragraph 5:*

“***Comparison with existing guidelines***
A comparison between our findings for diabetic patients with a CRC family history and established screening guidelines for individuals with a first-degree relative with CRC revealed a wide range of difference between our recommended risk-adapted starting ages of screening and those in the current guidelines (from –5 to 21 years) although the difference for other example ages could be even higher (S4 Table). Such a difference for diabetic patients without family history of colorectal cancer was 3-5 years depending on sex and benchmark ages of mass screening (Table 1).”

Minor comments
Page 11 the last sentence: I do not understand the intended meaning of the sentence.

*Response: That non-essential unclear sentence was deleted.*

Page 12: The starting age of screening in the UK is not 60.

*Response: Since flexible sigmoidoscopy is offered in England at around age 55, and not in all regions of the UK (*<https://www.gov.uk/guidance/bowel-cancer-screening-programme-overview#:~:text=Bowel%20cancer%20screening%20is%20offered,women%20in%20their%2056th%20year.>*), following sentences were amended in Results and Discussion:*

*Results, Subsection ‘Other benchmark ages (55 and 60)’, line 1:*

“As different countries have different benchmark ages for initiation of CRC mass screening in the population (ranging from 45 in the US to 55-60 in the UK), we provided risk-adapted starting ages of CRC screening for different benchmark ages (45, 50, 55, and 60 years; **Table 1**).”

*Discussion,* *last paragraph, line 1:*

“Since there is a wide disparity in CRC screening guidelines globally for age of first screening, such as age 55 in the Netherlands, age 55 or 60 in the UK (depending on location) …”

-----------------------------------------------------------

**References**

1.	Jensen OM PD, MacLennan R, Muir CS, Skeet RG. Cancer registration: principles and methods. IARC Sci Publ. 1991(95):1-288.

2.	Mukama T, Fallah M, Tian Y, Sundquist K, Sundquist J, Brenner H, et al. Risk-tailored starting age of breast cancer screening based on women's reproductive profile: A nationwide cohort study. Eur J Cancer. 2020;124:207-13.

3.	Mukama T, Kharazmi E, Xing X, Sundquist K, Sundquist J, Brenner H, et al. Risk-Adapted Starting Age of Screening for Relatives of Patients With Breast Cancer. JAMA Oncol. 2019.

4.	Tian Y, Kharazmi E, Brenner H, Xu X, Sundquist K, Sundquist J, et al. Calculating the Starting Age for Screening in Relatives of Patients With Colorectal Cancer Based on Data From Large Nationwide Data Sets. Gastroenterology. 2020;159(1):159-68 e3.

5.	Mukama T, Kharazmi E, Sundquist K, Sundquist J, Brenner H, Fallah M. Familial risk of breast cancer by dynamic, accumulative, and static definitions of family history. Cancer. 2020.

6.	Andersson DK, Svardsudd K, Tibblin G. Prevalence and incidence of diabetes in a Swedish community 1972-1987. Diabet Med. 1991;8(5):428-34.

7.	Thunander M, Petersson C, Jonzon K, Fornander J, Ossiansson B, Torn C, et al. Incidence of type 1 and type 2 diabetes in adults and children in Kronoberg, Sweden. Diabetes Res Clin Pract. 2008;82(2):247-55.

8.	Midthjell K, Bjorndal A, Holmen J, Kruger O, Bjartveit K. Prevalence of known and previously unknown diabetes mellitus and impaired glucose tolerance in an adult Norwegian population. Indications of an increasing diabetes prevalence. The Nord-Trondelag Diabetes Study. Scand J Prim Health Care. 1995;13(3):229-35.

9.	Bullard KM, Cowie CC, Lessem SE, Saydah SH, Menke A, Geiss LS, et al. Prevalence of Diagnosed Diabetes in Adults by Diabetes Type - United States, 2016. MMWR Morb Mortal Wkly Rep. 2018;67(12):359-61.

10.	Mendis S, Davis S, Norrving B. Organizational update: the world health organization global status report on noncommunicable diseases 2014; one more landmark step in the combat against stroke and vascular disease. Stroke. 2015;46(5):e121-2.

11.	World Health Organization. World health statistics overview 2019: monitoring health for the SDGs, sustainable development goals: World Health Organization.; 2019.

12.	NORDCAN: Cancer Incidence, Mortality, Prevalence and Survival in the Nordic Countries, Version 8.2 (26.03.2019). [Internet]. Danish Cancer Society. Accessed on 23/08/2020. 2019 [cited 23/08/2020]. Available from: [http://www.ancr.nu/](http://www.ancr.nu).

13.	Tian Y, Kharazmi E, Sundquist K, Sundquist J, Brenner H, Fallah M. Familial colorectal cancer risk in half siblings and siblings: nationwide cohort study. BMJ. 2019;364:l803.
