# Supplementary material for: Risk of colorectal cancer in patients with diabetes mellitus: A Swedish nationwide cohort study
Source: PLoS Med. 2020 Nov 13;17(11):e1003431. doi: 10.1371/journal.pmed.1003431 (PMC7665813; doi:10.1371/journal.pmed.1003431)
Supplement: Attachment — Submitted filename: R2 Response letter 8Oct2020.docx [file pmed.1003431.s010.docx]

**Author Response Letter to editors and reviewers’ comments**

**Requests from the editors:**

Title- please amend to "Risk of colorectal cancer among patients ..." and add Sweden in the title

*Response: The title was accordingly changed to:*

“Risk of colorectal cancer in patients with diabetes mellitus: A Swedish nationwide cohort study”

Short title and throughout please amend “diabetic patients” to patients with Type 2 diabetes

*Response: All instances of “diabetic patients” were amended to “patients with diabetes”.*

*We could not change all instances of “diabetes” to “Type 2 diabetes” as it would make a bias because we did not conduct stratified analyses by type due to lack of reliable information on type of diabetes, which has been mentioned as a limitation in Abstract and Discussion sections:*

*Abstract (last line of Methods and Findings section): “*The study limitations include lack of detailed information on diabetes type, lifestyle factors, and colonoscopy.*”*

*Discussion (paragraph 6, line 1):*

*“*We could not stratify our analyses by diabetes type because the ICD codes for diabetes diagnosis in our dataset did not accurately differentiate the type of diabetes until 1997 (ICD-10) and even after that the majority had both diagnoses, which might correspond to older definition of insulin-dependent and non-insulin-dependent diabetes mellitus rather than the actual type of diabetes.*”*

Abstract methods and findings- the first sentence needs some revision, perhaps “was carried out” or similar to end the sentence.

*Response: “was carried out” was added to the sentence and it reads now as following:*

*“*A nationwide cohort study (follow-up:1964-2015) involving all Swedish residents born after 1931 and their parents was carried out using record linkage of Swedish Population Register, Cancer Registry, National Patient Register and Multi-generation Register.*”*

Abstract methods and findings- please provide participant demographics like age range, sex etc

*Response: Demographics age range and sex were added to following sentence in Abstract Methods and Findings:*

*“*Of 12,614,256 individuals who were followed between 1964 and 2015 (51% men; age range at baseline 0-107 years), 162,226 developed CRC and 559,375 developed diabetes.”

Abstract and throughout- please ensure you specify which type of diabetes you mean. If this is Type 2 or insulin dependent- please mention that.

*Response: As explained above, we could not specify type of “diabetes” in the text because we did not conduct stratified analyses by type due to lack of reliable information on type of diabetes, which has been mentioned as a limitation in Abstract and Discussion sections:*

*Abstract (last line of Methods and Findings section):*

*“*The study limitations include lack of detailed information on diabetes type, lifestyle factors, and colonoscopy.*”*

*Discussion (paragraph 6, line 1):*

*“*We could not stratify our analyses by diabetes type because the ICD codes for diabetes diagnosis in our dataset did not accurately differentiate the type of diabetes until 1997 (ICD-10) and even after that the majority had both diagnoses, which might correspond to older definition of insulin-dependent and non-insulin-dependent diabetes mellitus rather than the actual type of diabetes.*”*

Abstract-please provide brief details on the data sources used for this work. For example- which inpatient/outpatient register was used and which cancer dataset?

*Response: First sentence of Methods and Findings section in the Abstract was amended to:*

*“*A nationwide cohort study (follow-up:1964-2015) involving all Swedish residents born after 1931 and their parents was carried out using record linkage of Swedish Population Register, Cancer Registry, National Patient Register, and Multi-generation Register.*”*

Abstract methods and findings- please mention the statistical analyses undertaken and the last sentence of this section should explicitly outline 2-3 limitations of your study design/methodology. We suggest you add “The study limitations include … “

*Response: The following sentence about statistical analyses was added to Methods and Findings section in the Abstract:*

“Age-specific 10-year cumulative risk curves were used to draw conclusions about how many years earlier patients with diabetes reach the 10-year cumulative risks of CRC in 50-year-old men and women (most common age of first screening), which were 0.44% and 0.41%, respectively.”

*The last sentience of Methods and Findings section in the Abstract was amended to: “*The study limitations include lack of detailed information on diabetes type, access to lifestyle factors and colonoscopy data.*”*

Abstract conclusions-please avoid assertions of primacy by adding “to our knowledge” or similar

*Response: The conclusion of the abstract was amended to:*

“Using high-quality registers, this study is to our knowledge the first one that provides novel evidence-based information for risk-adapted starting ages of CRC screening for patients with diabetes, who are at higher risk of early-onset CRC than the general population.*”*

Abstract conclusions- Please remove the word “personalised” as it is a bit misleading

*Response: The word “personalized” was removed.*

Data availability- we understand that the nationwide data cannot be shared by study authors. Could you please provide names and links to various data sources used in this study, noting that these cannot be shared by study authors but that interested parties may be able to request access by contacting the relevant persons. Please add contact details for the same. In addition, please note study authors cannot act as sole gatekeepers to datasets.

*Response: The following statement was added to the Metadata:*

*“This study made use of the Swedish Cancer Registry as well the Swedish National Patient Register. Data from these registers cannot be shared by study authors. However, further information and relevant contact details can be found on: https://www.socialstyrelsen.se/en/statistics-and-data/registers/register-information/*

*Links and email addresses for registers:*

*https://www.socialstyrelsen.se/en/statistics-and-data/registers/register-information/swedish-cancer-register/*

*(cancerregistret@socialstyrelsen.se)*

*https://www.socialstyrelsen.se/en/statistics-and-data/registers/register-information/the-national-patient-register/*

*(patientregistret@socialstyrelsen.se)”*

Author byline- we do not require author designations here, please remove all iterations of “group leader” “PhD students” etc

*Response: Author designations were removed.*

Author summary- this sentence requires simplification “Diabetic patients were observed to reach the population level of risk, at which screening starts, about 5 years earlier, and accordingly could start counseling for colorectal cancer screening earlier than the general population”. Please describe the analyses undertaken, in general language for accessibility to a general readership.

*Response: The analyses undertaken was added and statement was amended to:*

- “For each single age, we calculated the risk of developing colorectal cancer in the next 10 years; for example, at age 50, which is the most common age for starting colorectal cancer screening, the risk of developing colorectal cancer during next 10 years (age 50 to 59) in the Swedish population was 0.44% in men and 0.41% in women.
- Men and women with diabetes reached the risk levels for 50-year-old individuals (0.44% and 0.41%, respectively) at age 45 instead of age 50, i.e. 5 years earlier than the general population, whereas patients with an additional family history of colorectal cancer reach these screening risk thresholds, 12-21 years earlier than the general population.”

Author summary section “what did the researchers do and find” points 3 and 4 go considerably beyond what you may include in this section as it touches on application of your work. Please remove these points.

*Response: Points 3 and 4 were removed.*

Throughout- please use square brackets for references

*Response: All brackets used for references were amended to square brackets.*

Page 5, please add to our knowledge before “which resulted in the world’s largest and most robust study of its kind”.

*Response: The sentence was amended to:*

“, which resulted in, to the best of our knowledge, the world’s largest and most robust study of its kind.”

Page 8 first paragraph- please reword “averaged” to “average of” or other suitable alternatives

*Response: The term “averaged” was revised to “average of the” and the sentence now reads as:*

“For instance, for the 10-year cumulative risk at age 30, the average of the 10-year cumulative risks at ages 29, 30, and 31 was used, while for age 31, the average of the 10-year cumulative risks at ages 30, 31 and 32 was used and so on.”

Throughout the submission where you say “nationwide” please specify which country. When giving specific numbers of years of earlier disease onset should include "in the Swedish population

*Response: For each instance of “nationwide” the sentence was changed to specify Sweden or the Swedish population. Also, when giving specific numbers of years of earlier disease onset we included* "in the Swedish population.”

Page 15 – please rephrase “ poor lifestyle choices” to be specific and not stigmatising

*Response: The sentence containing “poor lifestyle choices” was amended to:*

“Type 2 diabetes and CRC share several risk factors including obesity and lack of regular physical activity.

Page 15- please replace “alcoholism” with high alcohol intake or similar

*Response: “alcoholism” was changed to “alcohol use disorder” in the text and in new Tables 1 and 2.*

RECORD checklist- please use paragraphs and sections instead of page numbers as these are likely to change

*Response: The revised RECORD checklist contains paragraphs and section.*

Please discuss the current level of adherence to CRC screening in the discussion and how this affects your results as well as potential implications for application.

*Response: Following sentences were added to the Discussion section, paragraph 7, line 7:*

“The lack of CRC screening data also did not have a significant impact on our findings and the potential implication of their application. This is because a nationwide organized CRC screening does not exist in Sweden. An organized screening as an official recommendation (not a law) has been introduced only as a pilot phase in 2008 in the Swedish Stockholm Gotland area merely for age 60-69, where even invitational coverage accounted for less than 9% of the nationwide screening-eligible population (age 50-74) [1].”

Please move Suppl tables 1 and 2 to the main manuscript

*Response: These tables have been added to the main manuscript as Tables 1 and 2 and cited appropriately. Previous Table 1 was renamed to Table 3 and cited accordingly.*

Overall all conclusions need to be tempered, such as "... reached the same level of CRC risk" as this is an observational study. In the same vein, please add limitations of your work, including information on potential confounders.

*Response: The conclusions have been tempered and apart from Discussion section, limitations were also added to the Abstract.*

Comments from Reviewers:

Reviewer #1: The authors have addressed our previous concerns sufficiently. Figures 1 & 2 might also show the case for No DM but with CRC family history, since that combination appears to be missing.

*Response: The curves for No DM but with CRC family history were added to Figures 1 and 2.*

**Reference in response letter** (Note: Reference number is different from the number in the main text, which is automatiicaly made by EndNote)

1.	Basu P, Ponti A, Anttila A, Ronco G, Senore C, Vale DB, et al. Status of implementation and organization of cancer screening in The European Union Member States—Summary results from the second European screening report. International Journal of Cancer. 2018;142(1):44-56. doi: 10.1002/ijc.31043.
